# A Conformational Switch in the Active Site of BT_2972, a Methyltransferase from an Antibiotic Resistant Pathogen *B. thetaiotaomicron*

**DOI:** 10.1371/journal.pone.0027543

**Published:** 2011-11-28

**Authors:** Veerendra Kumar, J. Sivaraman

**Affiliations:** Department of Biological Sciences, National University of Singapore, Singapore, Singapore; MRC National Institute for Medical Research, United Kingdom

## Abstract

Methylation is one of the most common biochemical reactions involved in cellular and metabolic functions and is catalysed by the action of methyltransferases. *Bacteroides thetaiotaomicron* is an antibiotic-resistant bacterium that confers resistance through methylation, and as yet, there is no report on the structure of methyltransferases from this bacterium. Here, we report the crystal structure of an AdoMet-dependent methyltransferase, BT_2972 and its complex with AdoMet and AdoHcy for *B. thetaiotaomicron VPI-5482* strain along with isothermal titration calorimetric assessment of the binding affinities. Comparison of the *apo* and complexed BT_2972 structures reveals a significant conformational change between open and closed forms of the active site that presumably regulates the association with cofactors and may aid interaction with substrate. Together, our analysis suggests that BT_2972 is a small molecule methyltransferase and might catalyze two O-methylation reaction steps involved in the ubiquinone biosynthesis pathway.

## Introduction

Methyltransferases (EC 2.1.1) comprise a group of approximately 140 transferase enzymes that catalyze the transfer of a methyl group from a universal donor molecule, such as *S*-adenosyl-L-methionine (AdoMet), to a target substrate acceptor molecule, such as DNA, RNA, proteins, lipids, or other small molecules and leave the by-product S-adenosyl homocysteine (AdoHcy). These enzymes play a crucial role in numerous biochemical processes, including signal transduction, biosynthesis, metabolism, protein modification, gene silencing, and chromatin regulation [1], [2]. Methyltransferases are structurally categorized into five classes (I–V). Whilst class I methyltransferases differ in their overall structure and substrate binding domain, all of the AdoMet-dependent methyltransferases in class I share a common Rossmann-fold at their catalytic site [3].

*Bacteroides thetaiotaomicron* is a gram-negative anaerobic bacterial pathogen with extreme disease-causing potential and antibiotic resistance. Predominantly found in the human intestinal tract [4], these bacteria dominate over other bacterial species and are involved in the uptake and degradation of otherwise non-digestible polysaccharides (e.g. amylose, amylopectin and pullulan), as well as in capsular polysaccharide biosynthesis, environmental sensing, signal transduction and DNA mobilization [4], [5]. The complete genome of the strain *B. thetaiotaomicron VPI-5482* has been sequenced [4] and an open reading frame (ORF) encodes a protein BT_2972 (accession no NP_811884) that is predicted to have a conserved AdoMet binding domain, which is a characteristic of most methyltransferases [6].

As a continuation of our studies toward understanding the structure and function of methyltransferases, we report here crystal structures of BT_2972, and the thermodynamics of the AdoMet/AdoHcy ligand binding. This study reveals significant conformational changes in a loop in the region of the active site (Glu121–Ile127), resulting in open and closed forms of the active site. In addition, our analysis suggests that BT_2972 is a small molecule methyltransferase, and may be involved in catalyzing the O-methylation reaction in the ubiquinone biosynthesis pathway.

## Materials and Methods

### Cloning and protein purification

The BT_2972 gene was cloned into expression vector pGS21a (GeneScript, USA) and the recombinant plasmid was transformed into *E. coli BL21 (DE3)* competent cells and plated onto ampicillin-containing agar plates [7]. Subsequently, a single colony was picked and used for large scale protein over-expression. The recombinant protein contains a noncleavable (His)_6_ tag for affinity purification. The protein was purified to homogeneity using a two-step procedure involving Ni^2+^-NTA affinity [8] and gel filtration chromatography in a buffer consisting of Tris-HCl (pH 8.0) and 200 mM NaCl. Prior to crystallization, the homogeneity of BT_2972 was verified by dynamic light scattering (DLS) experiments.

### Crystallization and structure determination

Crystallization trials of BT_2972 at a concentration 10 mg/ml, with and without AdoMet and AdoHcy (protein∶ligand concentration ratio 1∶5) were performed using commercially available screens from Hampton Research (Aliso Viejo, CA, USA), Jena Bioscience (Jena, Germany), Emerald BioSystems (WA, USA) and Qiagen (Valencia, CA, USA) by hanging drop vapour diffusion at room temperature (24°C). Initial conditions were further optimized and diffraction quality crystals of *apo* BT_2972 were obtained from a reservoir solution consisting of 0.12 M magnesium acetate and 16% (w/v) PEG3350, while the crystals of AdoMet and AdoHcy complexes were each grown from 25% (v/v) 2-propanol, 0.1 M MES monohydrate (pH 6.0) and 18% (w/v) polyethylene glycol monomethyl ether 2,000, respectively. Crystals were cryo-protected with 10% glycerol supplemented with reservoir solution and flash cooled in a cold N_2_ stream at 100 K [9].

Diffraction data sets for *apo* BT_2972 were collected with the Bruker AXS X8 Proteum X-ray system (wavelength 1.5418 Å) (Bruker AXS Inc., Madison, USA), while data for the AdoMet and AdoHcy complexes was collected at the beam line 13B1 (wavelength 1.000 Å) at the National Synchrotron Radiation Research Centre (NSRRC), Taiwan. All data sets were collected at 100 K and were indexed, integrated and scaled using HKL2000 [10]. While we were in the data collection stage, native protein coordinates were made available in the PDB database by Northeast Structural Genomics Consortium (PDB code 3F4K), but were not yet reported in the literature. Thus, the molecular replacement method was used to solve the structure of BT_2972 using the program Molrep-auto MR in CCP4 suite [11]. The molecular replacement solution clearly indicated the expected number of molecules in the asymmetric unit of BT_2972, predicted based on the Matthew's constant. The initial R-factors of the unrefined models were in the range of 0.39–0.42 with a correlation coefficient of ∼0.6. When required, protein models were manually built using the program COOT [12] and refinement performed using the program CNS [13]. Difference maps were calculated to position the ligands. At the final stage of the refinement, well-ordered water molecules were included. The models have good stereochemistry, with all residues within the allowed region of Ramachandran plot as analyzed by PROCHECK [14]. All structure-related figures reported were generated using PyMol [15].

### Isothermal titration calorimetry

The binding of AdoMet and AdoHcy to BT_2972 was studied using isothermal titration calorimetry [16]. Protein and stock solutions of AdoMet and AdoHcy were kept in a buffer consisting of 20 mM Tris-HCl (pH 8.0) and 200 Mm NaCl. The ITC experiments were performed using a VP-ITC calorimeter (Microcal, LLC) at 24°C with 0.4 ml of AdoMet or AdoHcy in the injector cell and 1.8 ml of protein in the sample cell. All samples were thoroughly degassed and centrifuged to remove precipitates. 10 µl injection volumes were used for all experiments. Two consecutive injections were separated by 5 min to reset the baseline. The control experiment, consisting of titration of AdoMet/AdoHcy against buffer, was performed and subtracted from each experiment to adjust for the heat of dilution of ligands (Figure S3). ITC data were analyzed with a single site fitting model using Origin 7.0 software (OriginLab Corp.).

### PDB deposition

The coordinates and structure factors of *apo* BT_2972, and AdoMet and AdoHcy with complexes were deposited in the PDB database [17] with accession codes 3SVZ, 3SXJ and 3T0I, respectively.

## Results

### BT_2972 sequence analysis

**Figure 1:**
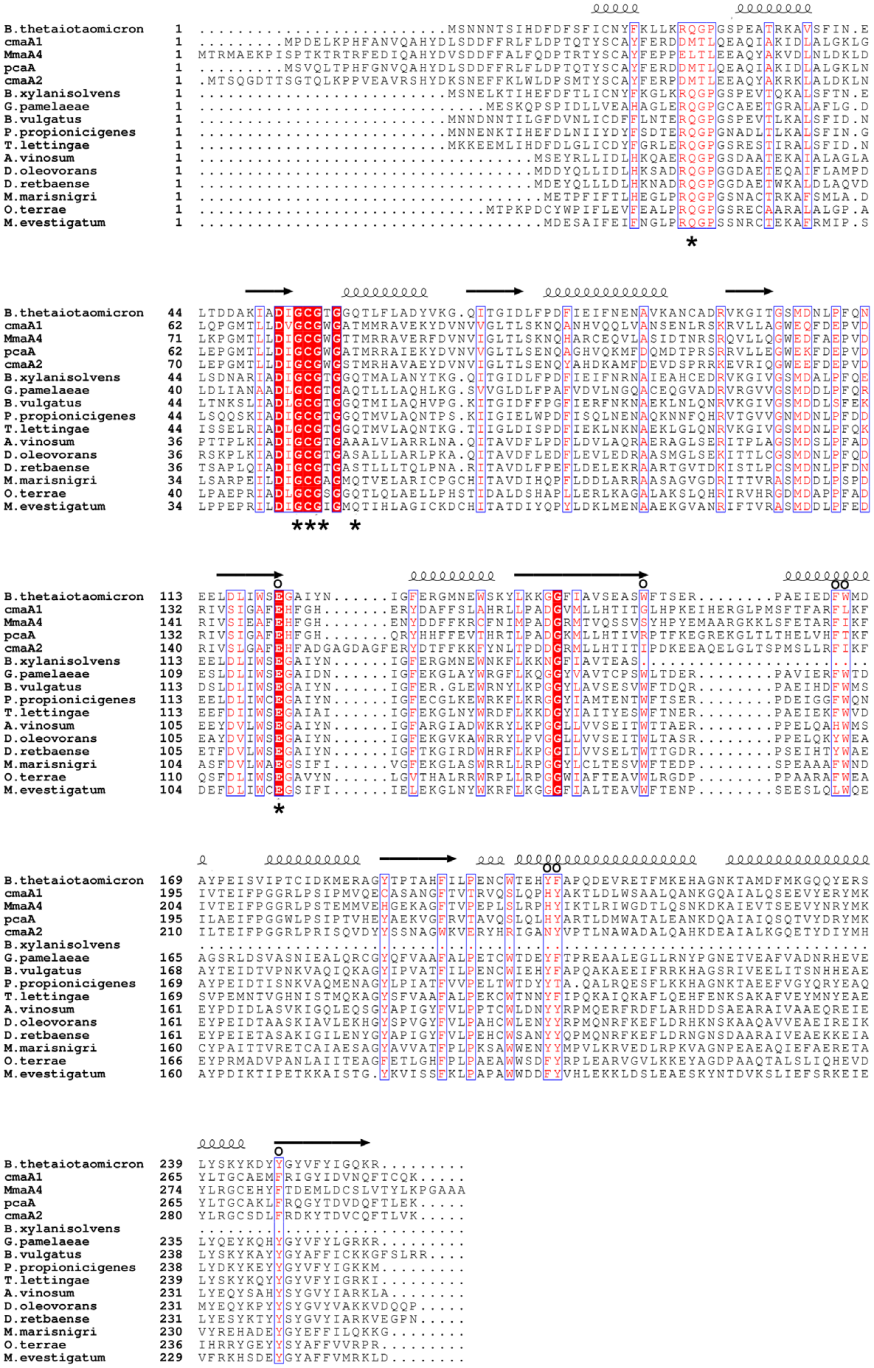
BT_2972 sequence analysis. Multiple sequence alignment of BT_2972 with selected sequences of methyltransferase of ubiquinone/menaquinone biosynthesis pathway and mycolic acid modifying methyltransferases. The top five sequences (1–5) show the structure based sequence alignment of BT_2972 with mycolic acid methyltransferases (CmaA1, PDB code 1KPG; MmaA4, PDB code 2FK8; PcaA, PDB code 1L1E and CmaA2, PDB code 1KPI). Sequences 6–16 represent the sequence based alignment of BT_2972 with the ubiquinone/menaquinone methyltransferases (*B. xylanisolvens* - CBK67164.1, *G. pamelaeae*- CBL04788.1, *B. vulgatus* -YP_001300506.1, *P. propionicigenes*- YP_004041425.1, *T. lettingae* -YP_001471184.1, *A. vinosum*- YP_003442314.1, *D. oleovorans* -YP_001528472.1, *D. retbaense* - YP_003197230.1, *M. marisnigri*- YP_001046804.1, *O. terrae*- YP_001818708.1, *M. evestigatum*- YP_003726621.1). Sequence similarities are highlighted in red, whereas sequence identities are shown as white letters on a red background. The residues that interact with AdoMet/AdoHcy (asterisks) and with substrate CTAB (open circle) are marked. The structure based alignment was obtained using DALI [23]. Alignment was carried out using ClustalW [31]. The secondary structure for BT_2972 is shown on the top. This diagram was generated using the program ESPript [32]. Abbreviation- B: *Bacteroides*, G: *Gordonibacter*, P: *Paludibacter*, T: *Thermotoga*, A: *Allochromatium*, D: *Desulfococcus*, M: *Methanoculleus*, O: *Opitutus*.

A PSI-BLAST [18] search with the BT_2972 sequence indicated that it belongs to the AdoMet-dependent methyltransferase family. In particular it revealed 80 and 51% sequence identity to two methyltransferases involved in the ubiquinone/menaquinone biosynthesis pathways of *Bacteroides xylanisolvens XB1A* (score = 259 and E-value = 2e^−67^) and *Gordonibacter pamelaeae 7-10-1-b* (score = 266 and E-value = 2e^−69^), respectively. The PSI-Blast e-value indicates that these two sequences are evolutionarily related to BT_2972. Sequence alignment of the cluster of orthologous groups (COG) of similar ubiquinone/menaquinone methyltransferase (COG2226H) revealed several highly conserved residues [19] (Figure 1). In particular the AdoMet/AdoHcy and the substrate binding site as well as the interacting residues are highly conserved (Figure 1). The ubiquinone and menaquinone pathways share a common intermediate and common methyltransferase.

### Structure of BT_2972 and its AdoMet/AdoHcy complexes

**Figure 2:**
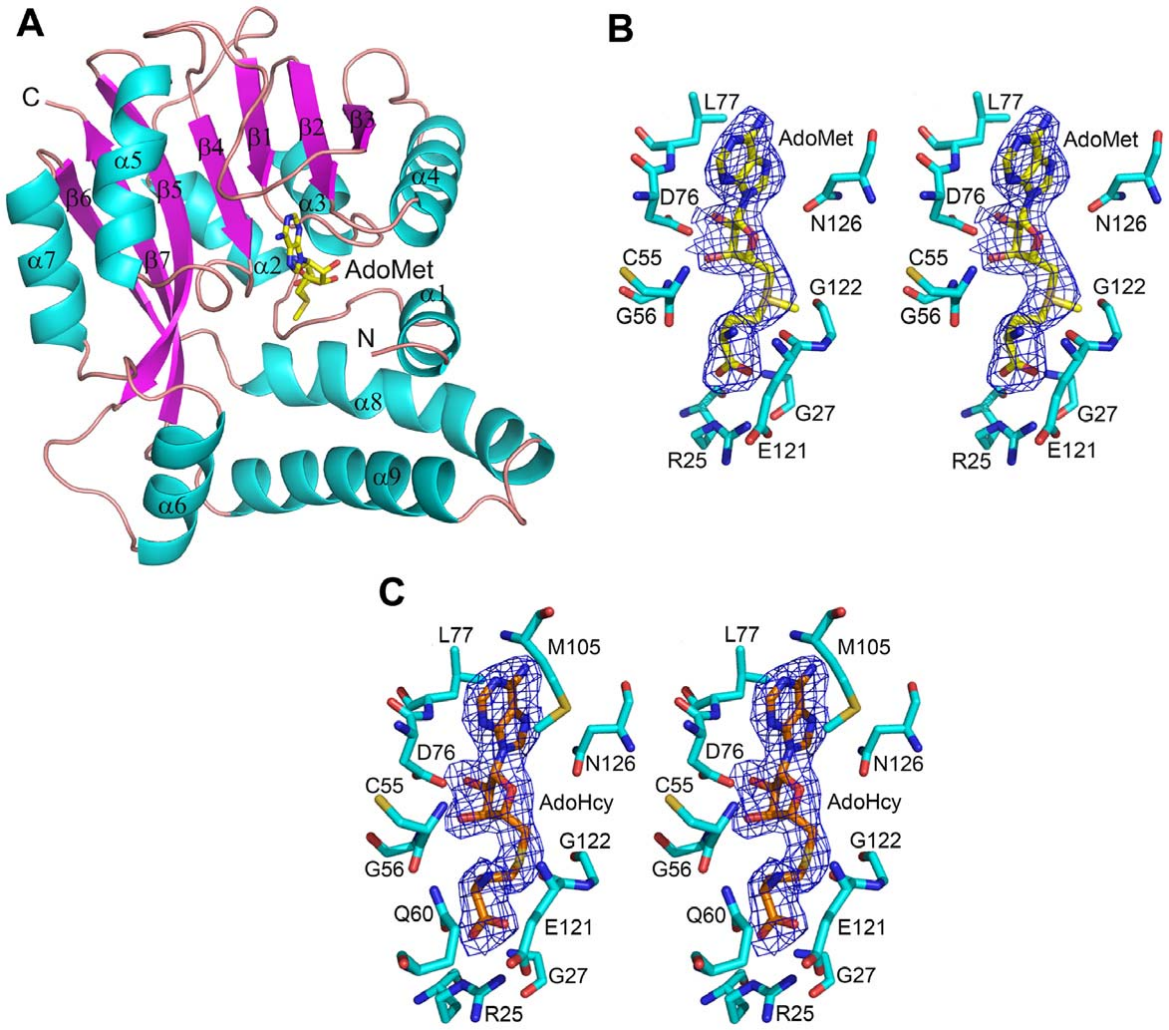
Structure of BT_2972. (**A**) Ribbon representation of the crystal structure of BT_2972-AdoMet complex. The α helices and β sheets are shown in cyan and magenta, respectively. The N- and C- termini and the secondary structural elements are labelled. The AdoMet is shown in stick representation (yellow). The structure of *apo* BT_2972 and BT_2972-AdoHcy are provided in the supplementary section (Figure S1). Stereo view of *2Fo-Fc* map of (**B**) AdoMet and (**C**) AdoHcy in complex with BT_2972. The map is contoured at a level of 1.0σ. AdoMet is shown in yellow (**B**) and AdoHcy is shown in orange (**C**). Selected interacting residues from BT_2972 are shown in cyan colour.

Crystal structure of the *apo* BT_2972 and its complexes with AdoMet and AdoHcy were solved and refined up to a resolution of 2.9, 2.5 and 2.4 Å, respectively (Table 1, Figure 2, and Figure S1). The BT_2972 structures (*apo*, AdoMet and AdoHcy complexes) and the search model (PDB code 3F4K) all crystallized in three different space groups. BT_2972 crystallized with two molecules in the asymmetric unit (Table 1), while the 3F4K crystal has one molecule in the asymmetric unit. Notably, each of the reported structures crystallized in different conditions. The pairwise comparison of these structures did not reveal any major conformational changes other than to the active site loop region, residues Glu121-Ile127. 3F4K is similar to BT_2972 *apo* form (RMSD 0.7 Å for all Cα atoms). Despite the presence of two molecules of BT_2972 in the asymmetric unit, gel filtration and dynamic light scattering (DLS) experiments showed that they exist as monomers in solution. The BT_2972 construct has a total of 257 amino acids and a (His)_6_-tag at the N-terminus. In all three models, the electron density map for the first 12 amino acids and His-tag was not well-defined and, as such, these residues were not included in the model.

**Table 1:** Crystallographic data and refinement statistics.

	Native	AdoMet	AdoHcy
Cell parameters (Å, °)	a = 60.55b = 74.38c = 117.11	a = 120.79b = 59.68c = 77.62β = 104.61	a = 60.64b = 74.52c = 117.22
Space group	P 2_1_ 2_1_ 2_1_	C 2	P 2_1_ 2_1_ 2_1_
Data collection			
Resolution range (Å) *	50.0 – 2.9 (3-2.9)	50.0 - 2.5 (2.59-2.5)	50.0 - 2.4 (2.49-2.4)
Wavelength (Å)	1.5418	1.00	1.00
Observed reflections >1σ	93702	58009	73707
Unique reflections	11731	18262	20346
Completeness (%)	95.8 (91)	97.4 (86.9)	94.5 (91.0)
Overall (I/σ (I))	10.6 (3.5)	18.7 (2.6)	15.5 (2.9)
R_sym_ a	0.109 (0.177)	0.058 (0.192)	0.071 (0.215)
Refinement and quality^b^			
Resolution range (Å)	50.0 – 2.9	50.0 - 2.5	50.0 - 2.4
R_work_ c (no. of reflections)	0.20 (8952)	0.23 (13792)	0.23 (16142)
R_free_ d (no. of reflections)	0.26 (674)	0.27 (1013)	0.26 (1189)
RMSD bond lengths (Å)	0.008	0.008	0.007
RMSD bond angles(°)	1.17	1.12	1.00
Average B-factors^e^ (Å^2^)			
Main chain	22.1	47.1	32.0
Side chain	25.2	48.2	34.4
Ramachandran plot			
Most favored regions (%)	91.0	90.1	92.1
Additional allowed regions (%	8.5	9.6	7.9
Generously allowed regions (%)	0.5	0.4	0.0
Disallowed regions (%)	0.0	0.0	0.0

a R_sym_ = ∑|I_i_–<I>|/|I_i_| where I_i_ is the intensity of the ith measurement, and <I> is the mean intensity for that reflection.

b Reflections with I>σ was used in the refinement.

c R_work_ = |F_obs_–F_calc_|/|F_obs_| where F_calc_ and F_obs_ are the calculated and observed structure factor amplitudes, respectively.

d R_free_ = as for R_work_, but for 5% of the total reflections chosen at random and omitted from refinement.

e Individual B-factor refinements were calculated.

*The high resolution bin details are in the parenthesis.

The BT_2972 molecule is a single domain, globular protein comprising both the cofactor and substrate binding site within the same domain (Figure 2A). It consists of a total of nine α-helices and seven β-strands of different lengths that are distributed throughout the protein sequence. The core domain adopts a typical class I Rossmann-like AdoMet-binding fold that is common to all class I AdoMet-dependent methyltransferases, comprising a seven-stranded β-sheet (β3↓β2↓β1↓β4↓β5↓β7↑β6↓) flanked by three α helices on each side [20]. In this fold, all β-strands in the active site are planar and parallel to each other, with the exception of the anti-parallel strand β7. The AdoMet/AdoHcy binding region is primarily located within the N-terminal region (Figure 2A), while the substrate is proposed to bind to C-terminal residues. Depending on the target for methylation, substrate binding sites of different methyltransferases vary in structure and size to accommodate the different targets.

### Thermodynamics of AdoMet/AdoHcy binding

**Figure 3:**
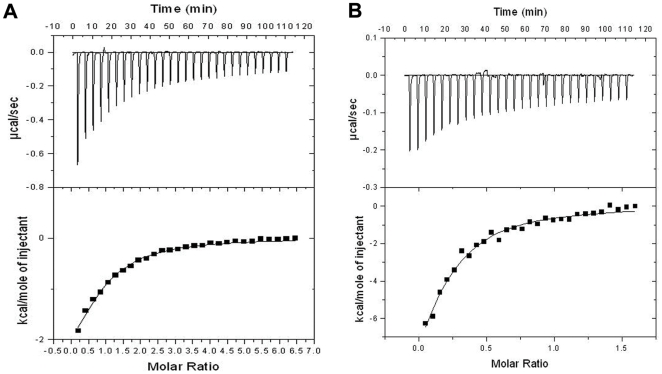
ITC profiles for BT_2972 titrated against the cofactors (A) AdoMet and (B) AdoHcy. The raw ITC data for injections of ligands into the sample cell containing the native protein are shown in the upper panels of ITC profiles. The peaks were normalized to the ligand∶protein molar ratio and were integrated as shown in the bottom panels. Solid dots indicate the experimental data, and their best fit was obtained from a nonlinear least squares method, using a one-site binding model (depicted by a continuous line).

The interactions between BT_2972 with AdoMet and AdoHcy were studied using ITC (Figure 3). As predicted from the crystal structure, ITC experiments showed a single site binding model for both AdoMet and AdoHcy. The thermodynamic binding parameters (where K_a_ is the association constant, ΔH is the change in enthalpy, and N is the number of binding sites) were calculated from ITC data fitting, as follows: for AdoMet: K_a_ = 0.395×10^5^ M^−1^ (±0.0435×10^5^), ΔH = −3.775 kcal/mol (±0.42), N = 0.78.±0.07; and for AdoHcy: K_a_ = 3.498×10^5^ M^−1^ (±0.66×10^5^), ΔH = −18.63 kcal/mol (±5.6), N = 0.82±0.04. Although AdoMet and AdoHcy have similar interactions with BT_2972 in terms of the intermolecular contacts in the crystal, the affinity between the ligands and BT_2972 differs by a factor of ten in K_a_. The binding affinity of AdoMet to BT_2972 is comparable to its nearest structural homolog BVU_3255 from *B. vulgatus ATCC 8482* (∼59% sequence identity). However AdoHcy binding to BT_2972 is approximately twelve-fold stronger than that for BVU_3255 [21].

### AdoMet/AdoHcy binding pocket of BT_2972

BT_2972 was crystallized with and without AdoMet and AdoHcy (Figure 2 and Figure S1B). In the active site, both ligands are buried and are oriented in a similar way, with their adenosyl base moiety facing outside and the carboxypropyl moiety facing inside the molecule. Residues located in the active site region, such as Arg25, Gln26, Gly27, Gly54, Cys55, Gly56 and Gln60 interact with the carboxypropyl moiety of AdoMet and AdoHcy in both complexes. Similarly, residues within the region of the active site, such as Leu77, Gly103, Met105, Glu121 Gly122 and Asn126, interact with the adenosyl moiety of the ligands (Figure 2B and 2C). These residues line up in the active site, and all interact with the ligands either via hydrophobic or hydrogen bonding contacts (Figure 2B and 2C). There are ten hydrogen bonding contacts (<3.3 Å) between the ligand and BT_2972 in each complex. The superposition of these two complex structures gave a root mean square deviation (RMSD) of 0.4 Å. The carboxyl group of Asp76 in β2 strand was found to interact with the oxygen atom of the AdoMet/AdoHcy ribose ring. This is a conserved position with an Asp or Glu residue in a number of methyltransferases that interacts with the ribose ring of the bound AdoMet/AdoHcy through hydrogen bonding contacts [22].

### Conformational switch acts as a gate to the active site

**Figure 4:**
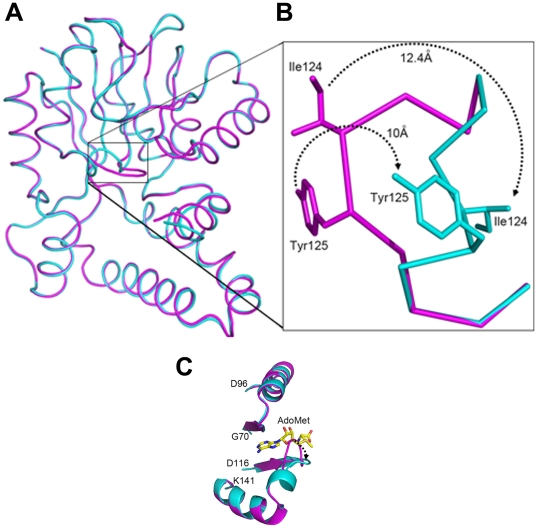
Conformational switch in the fragment Glu121-Ile127. A) The superposition of *apo* BT_2972 (magenta) and the BT_2972-AdoMet complex (cyan). The conformational change in the fragment Glu121-Ile127 is marked by a rectangular box. **B**). A close up view of the conformational change. **C**) Conformational change in the Glu121-Ile127 region with bound ligand (AdoMet is shown here).

**Figure 5:**
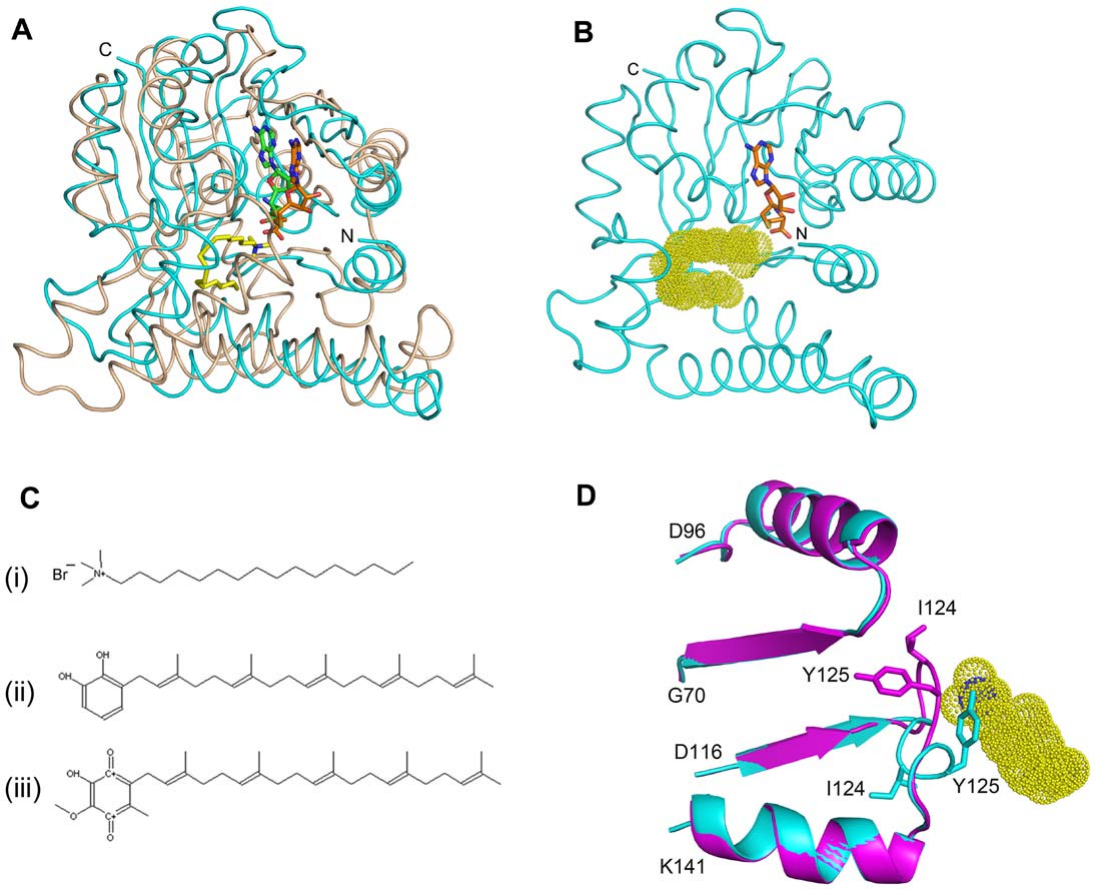
Interpretation of possible substrate binding site and substrate. **A**) Cα trace for the superposition of BT_2972-AdoHcy (cyan) and mycolic acid cyclopropane synthase CmaA1-AdoHcy-CTAB from *M. tuberculosis* (PDB code 1KPG) (light orange). AdoHcy occupies the same region in the active sites of the two proteins. The carboxypropyl moiety is lying in same plane but ribose and adenosyl moieties are displaced by 3.5 Å. The methylation substrate CTAB of CmaA1 is shown in stick representation in yellow. The alignment was carried out in PyMol [15]. **B**) The proposed substrate binding region of BT_2972-AdoHcy is shown as yellow dotted surface. The inferred substrate binding site also is shown as a surface diagram in Figure S5. **C**) Similarity between CTAB and the proposed substrate for BT_2972: (i) CTAB contains a 16-carbon-long alkyl chain with a positively charged quaternary ammonium group at one end. (ii) 2-polyprenyl-6-hydroxyphenol and (iii) 2-polyprenyl-3-methyl-5-hydroxy-6-methoxy-1,4-benzoquinone; both substrates have a 20-carbon long alkyl chain. **D**) Conformational change in the fragment Glu121-Ile127 with respect to the substrate binding site. Side chains of Ile124 and Tyr125 in AdoMet/AdoHcy complex structure project towards the substrate site and would likely enhance interactions with a hydrophobic substrate.

Superposition of the *apo* BT_2972 with AdoMet- and AdoHcy-bound structures gave a RMSD of 1.0 and 0.8 Å, respectively, for 245 Cα atoms (Figure 4A). A large conformational change was observed between the residues Glu121 and Ile127 upon the binding of AdoMet/AdoHcy in the active site region. This region is located in the loop between β4 and α5, with Glu121, Gly122 and Asn126 making direct contact with AdoMet/AdoHcy. Compared with the *apo* BT_2972, the backbone atoms of the AdoMet/AdoHcy complexes of this region are moved approximately 9 Å (with a maximum side chain movement of Ile124 by 12.4 Å and Tyr125 by 10 Å), opening up the active site (Figure 4B). The *apo* BT_2972 thus represents the closed form of the active site. In the *apo* structure, the loop Glu121-Ile127 protrudes into the cofactor binding region, with the side chains of Ile124 and Tyr125 occluding the active site (Figure 4C). However, on the other side of the active site cleft, there was no conformational change observed in the wall of the cleft, containing residues Leu77 and Phe81. This suggests that the loop region (Glu121-Ile127) acts as a flexible gate that enables the cofactors to enter or leave the active site region. In addition, this loop region may also enhance the hydrophobic interactions with substrate (Figure 5D).

### Structural comparison with other homologs

A search for structurally similar proteins in PDB database using the DALI server [23] revealed that BT_2972 shares highest homology with several class I Rossmann fold methyltransferases (RFM). The most structurally similar protein with known function that we identified is *Mycobacteriam tuberculosis* hydroxymycolate synthase MmaA4 (Hma; PDB code 2FK8; Dali score 20.4; RMSD 3.3 Å for 237 Cα atoms; sequence identity 12%), an AdoMet-dependent methyltransferase involved in the production of branched chain mycolic acid [24]. Next is cyclopropane mycolic acid synthase from *M. Tuberculosis* (Cma1; PDB code 1KPG, Dali score 19.9; RMSD 3.3 Å for 232 Cα atoms; sequence identity 12%) [25]. CmaA1 is an AdoMet-dependent protein with a similar α/β Rossmann fold that is involved in site-specific methylation of mycolic acid. Figure 5A shows the superposition of the structure of BT_2972-AdoHcy and CmaA1 bound to cetyltrimethylammonium bromide (CTAB) and AdoHcy [25]. We observe close alignment of the active site regions of these two structures including the bound cofactors (Figure 5A). The carboxypropyl portions of AdoMet in both proteins are in the same plane, but the ribose and adenosyl moieties show some deviation. The carboxypropyl moiety of AdoMet contains a ‘catalytic’ methyl group and this group is located next to methylation site of CTAB. The similarity in the active site and the position of the AdoMet methyl group suggests that these proteins might act on substrates with similar chemical properties.

### Possible Substrate Binding Site and Substrate

The superposition of CmaA1-CTAB-AdoHcy complex with our BT_2972-AdoHcy structure allowed us to propose the substrate binding site for BT_2972. The substrate CTAB occupies a space that presents as a cavity in the BT_2972 complex adjacent to the AdoMet/AdoHcy binding site, and it is reasonable to suggest that this region corresponds to substrate binding site in BT_2972 where methylation occurs (Figure 5B). The site primarily consists of C-terminal residues and is surrounded by helices α1, α6 and α9 (yellow dotted region in Figure 5B and Figure S4) of the active site region. Most of the amino acids are bulky and hydrophobic in nature and therefore suitable to interact with a substrate of a hydrophobic nature. The chemical properties of the CmaA1 substrate CTAB are similar to those of the ubiquinone pathway intermediates that undergo methylation, with long aliphatic chains (Figure 5C). This suggests that substrates for BT_2972 could be intermediates of the ubiquinone pathway, namely 2-polyprenyl-6-hydroxyphenol and 2-polyprenyl-3-methyl-5-hydroxy-6-methoxy-1, 4-benzoquinone. The hydrophobic environment and shape of the proposed substrate binding site of BT_2972 could potentially form a hydrophobic cluster with the incoming substrate. Moreover, in the open conformation, the loop (Glu121-Ile127) that is located between the AdoMet/AdoHcy binding site and the proposed substrate binding site would enhance the hydrophobic interactions with the bound substrate by bringing its own hydrophobic residues (Ile124 and Tyr125) into contact with the substrate (Figure 5D).

Figure 1 shows the comparison of sixteen MTase sequences. The top five sequences were aligned based on their structural similarity and the bottom eleven sequences, whose structures are not known, were aligned based on their sequences. These alignments show the conservation of key residues among these sequences and suggest a similar structure and active site for these proteins. Notably, the substrate binding site of CmaA1 and the proposed substrate binding site for BT_2972 are conserved. Several CTAB (substrate) interacting residues of CmaA1 are found conserved in BT_2972 such as Glu121 (Glu140), Trp166 (Leu192), Phe206 (Tyr232), and Tyr247 (Phe273). The residue numbers corresponding to CmaA1 are given in the parentheses. In addition several hydrophobic residues such as Trp153, Phe165, Tyr205 of BT_2972 are found conserved in the inferred substrate binding cavity of BT_2792. Moreover, this proposed substrate binding site in BT_2972 is close to the transferable methyl group of AdoMet, indicating that it fulfils the geometric requirements for S_N_2 methylation reactions: a linear arrangement of the substrate acceptor atom, the transferred methyl group and the sulphur atom of AdoMet to form the appropriate transition state. However the precise identification of the true substrate preference of BT_2972 remains to be experimentally determined.

## Discussion

Class I AdoMet-dependent methyltransferases consist of a well-conserved AdoMet-binding region that is responsible for cofactor binding and methylation and a highly variable substrate-binding region [26]. Macromolecule and small molecule methyltransferases have several distinguishing features in addition to their core Rossmann fold. Macromolecular methyltransferases have additional secondary structural elements at their C terminus [1] and in most cases, their substrate binding region is a separate domain used to engage with the substrate such as DNA, RNA or proteins. In contrast, small molecule methyltransferases lack these two major structural features, with the majority having two additional α helices at the N-terminus and others showing residue insertions between β5 and α7; and β6 and β7 [1].

Here, we report the crystal structure of the AdoMet-dependent methyltransferase BT_2972 from *B. thetaiotaomicron VPI-5482* strain which shows the following structural features: 1) there is no separate substrate binding domain; 2) there is no additional secondary structure at the C-terminus; 3) there are two small α helices (α1 and α2) at the N-terminus; and 4) there are helices between β5 and α7 (α6) and between β6 and β7 (α8 and α9), respectively (Figure 2A). Furthermore, *E. coli Bl21 (DE3)* harbouring the BT_2972 coding region was tested for resistance to different antibiotics (kanamycin, tetracyclins, erythromycin and chloroamphenicol). These antibiotics confer resistance by interfering with the function of macromolecules in the host cell. MTases add a methyl group in the target macromolecule at the specific antibiotic binding site, thereby disrupting the antibiotic binding, and conferring resistance to the antibiotics. Each of the antibiotics tested here retained their activity against this strain. This suggests that BT_2972 might not have a macromolecule substrate. Based on its specific structural features, sequence analysis and antibiotic resistance tests we suggest that BT_2972 is a small molecule methyltransferase. Moreover the structure of BT_2972 is similar to that of an uncharacterized ubiquinone/menaquinone methyltransferase available in the PDB: TM1389 from *Thermotoga maritime* MSB8 (PDB code 2AVN, Dali score 14.4; RMSD 3.3 Å for 179 Cα atoms; sequence identity 14%); whose structure is not yet fully described in the literature.

The ubiquinone pathway involves three methylation steps while menaquinone pathway has one, with chorismate as the common intermediate for both pathways [27]. The three steps of ubiquinone pathway are catalyzed by two methyltransferases encoded by the *UbiG* and *UbiE* genes (Figure S2). The *UbiE*-encoded methyltransferase is common between the two pathways and is highly conserved amongst the bacteria. In the case of *B. thetaiotaomicron VPI-5482*, BT_4216 is annotated as *UbiE* [4]. In contrast, the sequences for *UbiG*-encoded methyltransferases (ubiquinone biosynthesis AdoMet-dependent O-methyltransferase) are highly variable amongst the bacteria. This leads us to speculate that BT_2972 may represent the UbiG-encoded methyltransferase (the other methyltransferase of this pathway) in *B. thetaiotaomicron VPI-5482* that catalyzes the two O-methyltransformation reactions in this organism.

**Figure 6:**
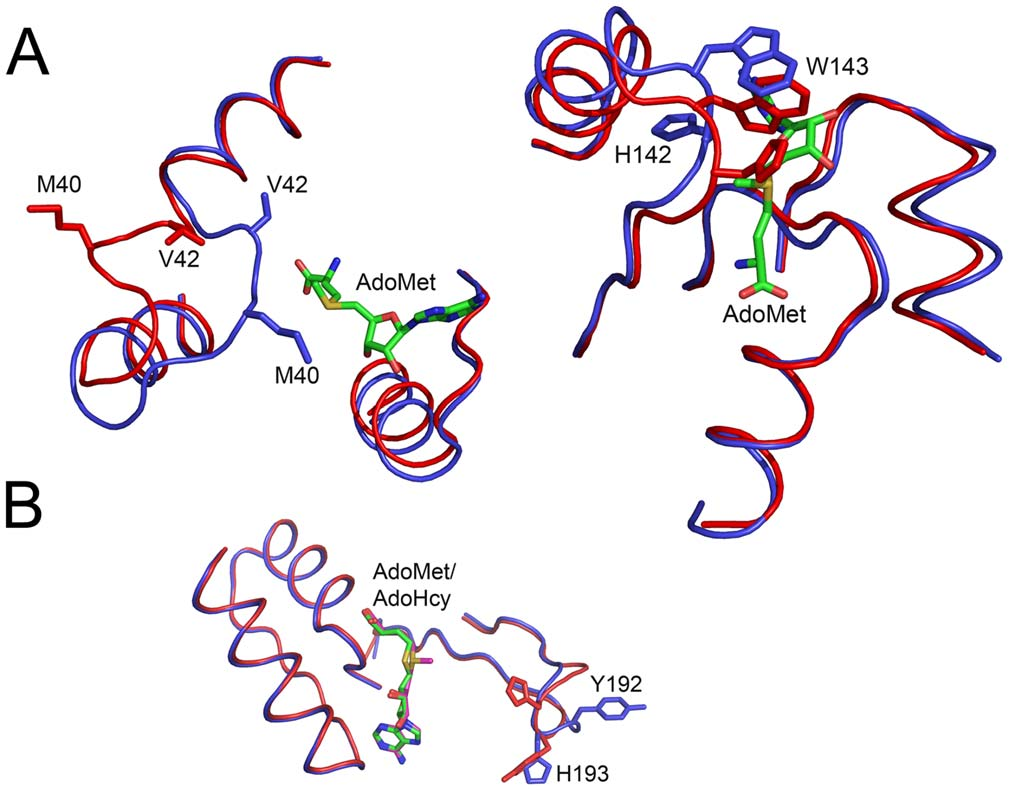
A Comparison of conformation change in some of the previously reported methyltransferases. A) Comparison of *apo* (red) and AdoMet-bound (blue) rat catechol-O-methyltransferase (PDB code 2ZLB and 1VIB, respectively). A large conformational change takes place in the loop Lys36-Val42 upon AdoMet binding that moves this loop towards the AdoMet (left panel). Also in the same structure a side chain movement was observed for the residue His 142 and Trp 143 (right panel). AdoMet is shown as stick representation in green. **B**) In L-isoaspartyl (D-aspartyl) methyltransferases (PDB codes 1JG1 and 1JG4), the Tyr192 and His193 side chains are rearranged. His193 points towards the cofactor when a catalytic methyl group is present on the cofactor (AdoMet, shown in red), whereas it points towards solvent when the methyl group is absent (AdoHcy, shown in blue). Similarly Tyr192 makes a 10 Å movement and a rotation of 90° between the AdoMet and AdoHcy complexes.

A key observation from our crystal structure in BT_2972 is the conformational change in the active site region upon cofactor binding which shows the opening and closing of the gate access to the active site. Conformational changes near the AdoMet-binding site have been observed in other methyltransferases, such as in rat catechol-O-methyltransferase [28], L-isoaspartyl (D-aspartyl) methyltransferases [29], betaine homocysteine S-methyltransferase [30]. In the case of rat catechol-O-methyltransferasee (PDB code 1VID and 2ZLB), the conformational change occurs in the backbone atoms of the loop Lys36-Val42 (∼12.0 Å) upon AdoMet binding close to the carboxylpropyl moiety of AdoMet. However, this loop does not occupy the AdoMet binding site, and only upon cofactor binding the backbone of this loop move towards the carboxyl moiety of AdoMet. Further, the side chains that occupy the AdoMet binding site in the *apo* structure (His142 and Trp143) move away (Figure 6A) to accommodate the incoming ligand [28]. In the case of L-isoaspartyl (D-aspartyl) methyltransferases (PDB codes 1JG1 and 1JG4) [29], only the side chains of Tyr192 and His193 flip outward between the AdoMet and AdoHcy complexes. In AdoMet-bound structure, these side chains project toward the cofactor; but in the AdoHcy complex, these side chains flip outward (Figure 6B). For the betaine homocysteine S-methyltransferase [30], the backbone and the side chain atoms of Phe76 and Tyr77 move upon substrate binding in comparison with its *apo* structure without interacting with the substrate (Figure S5). Unlike with these previous examples, the conformational change in BT_2792 appears important for ligand binding. The observed conformational change in the loop (Glu121-Ile127) in the active site is near to the ribose and adenosyl base binding region of the ligands, and is accomplished by the movement of backbone atoms up to 9 Å along with side chain movement up to 12 Å.

In summary, we report the crystal structures of an AdoMet-dependent methyltransferase BT_2972 from an antibiotic resistant bacterium *B. Thetaiotaomicron VPI-5482*. BT_2972 is the first representative structure of an AdoMet-dependent methyltransferase from *B. Thetaiotaomicron*. The comparison between the structures of the *apo* and AdoMet/AdoHcy complexes reveals that open/closed nature of the active site may regulate cofactor binding and substrate interactions. The isothermal titration calorimetric studies showed different binding affinities of BT_2972 for AdoHcy and AdoMet. The structural and sequence analyses suggest that BT_2972 is a small molecule methyltransferase that may catalyze two O-methylation reaction steps in the ubiquinone biosynthesis pathway.

## Supporting Information

Figure S1 **Ribbon representation of the crystal structure of (A)** ***apo*** **BT_2972 and B) BT_2972-AdoHcy complex.** The α helices and β sheets are shown in cyan and magenta colour, respectively. The N- and C-termini, and secondary structure elements are labelled. The AdoHcy is shown in a stick representation (orange).
(TIF)

Figure S2 **The schematic representation of the proposed biosynthesis of ubiquinone in bacteria.** There are three methylation reactions in this pathway catalyzed by UbiE and UbiG. This figure is prepared based on the literature with possible intermediates and possible enzymes involved in this biosynthesis [27], [33]. Abbreviations used in this diagram are – UbiC: chorismate–pyruvate lyase; UbiA: 4-hydroxybenzoate polyprenyltransferase; UbiD: 3-polyprenyl-4-hydroxybenzoate carboxy-lyase; UbiB: ubiquinone biosynthesis monooxygenase UbiB; UbiG: ubiquinone biosynthesis AdoMet-dependent O-methyltransferase; UbiH: ubiquinone biosynthesis monooxgenase; UbiE: ubiquinone/menaquinone biosynthesis methyltransferase; and UbiF: ubiquinone biosynthesis monooxgenase.
(TIF)

Figure S3 **The ITC control experiments.** **A**) Titration profile for AdoMet against buffer. A similar figure was obtained for AdoHcy titration against buffer. **B**) Titration of buffer against BT_2972 protein solution.
(TIF)

Figure S4 **The molecular surface representation of the inferred substrate binding site with respect to the bound AdoHcy is shown as yellow dotted region on the surface of the BT_2972-AdoHcy complex.**
(TIF)

Figure S5 **Figure shows the conformational change in betaine homocysteine S-methyltransferase upon substrate binding (PDB codes: 1UMY (from rat) and 1LT8 (from human)).** The *apo* protein is shown in red and S-(D-carboxybutyl)-L-homocysteine (CB-Hcy) complex is in blue. The backbone and the side chain atoms of Phe76 and Tyr77 are shifted in the transition-state analog (CB-Hcy) complex in comparison with the *apo* structure.
(TIF)
