# Supplementary figures and images for: A Conformational Switch in the Active Site of BT_2972, a Methyltransferase from an Antibiotic Resistant Pathogen B. thetaiotaomicron
Source: PLoS One. 2011 Nov 28;6(11):e27543. doi: 10.1371/journal.pone.0027543 (PMC3225368; doi:10.1371/journal.pone.0027543)

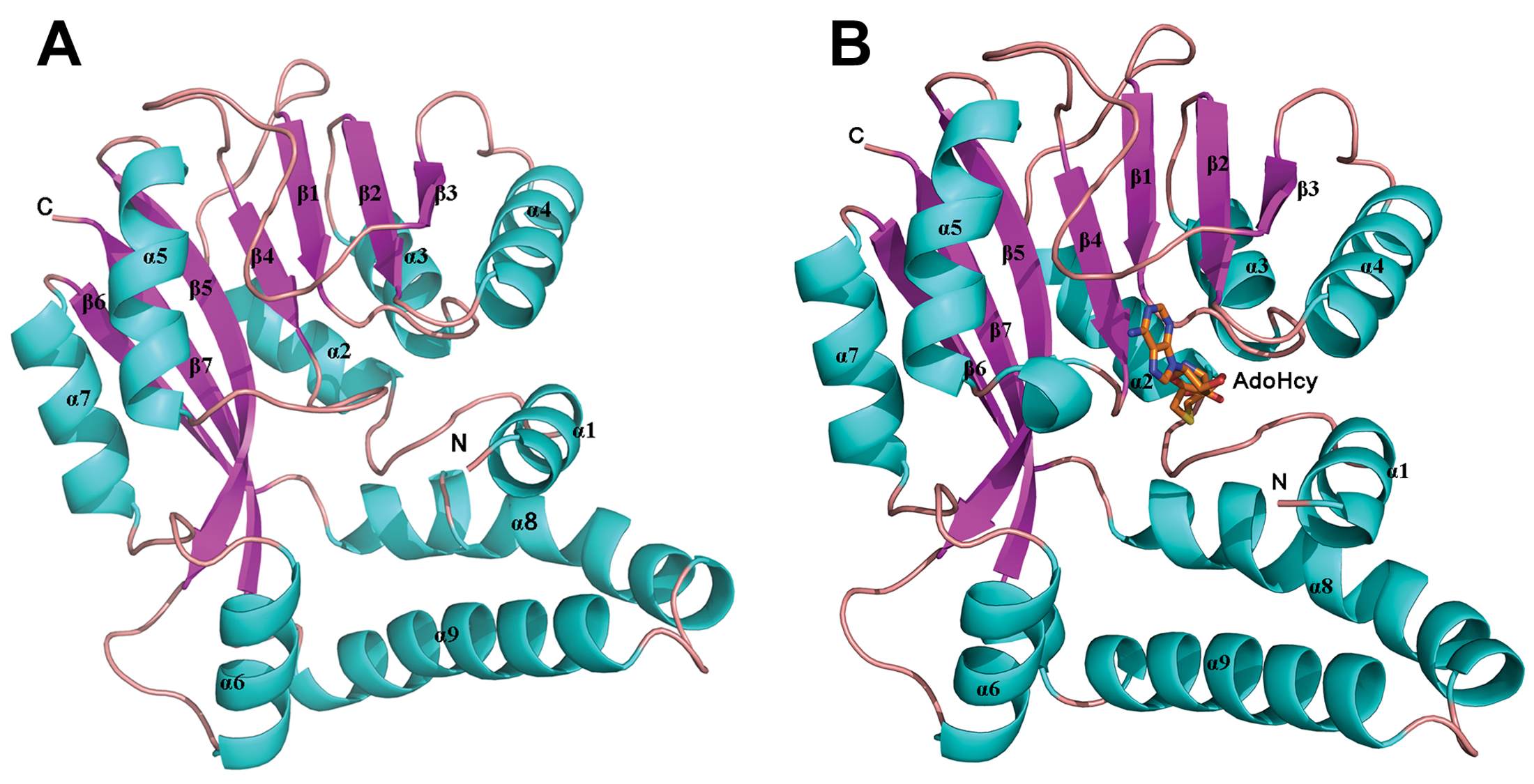

Supplement: Figure S1 — Ribbon representation of the crystal structure of (A) apo BT_2972 and B) BT_2972-AdoHcy complex. The α helices and β sheets are shown in cyan and magenta colour, respectively. The N- and C-termini, and secondary structure elements are labelled. The AdoHcy is shown in a stick representation (orange). (TIF) [file pone.0027543.s001.tif]

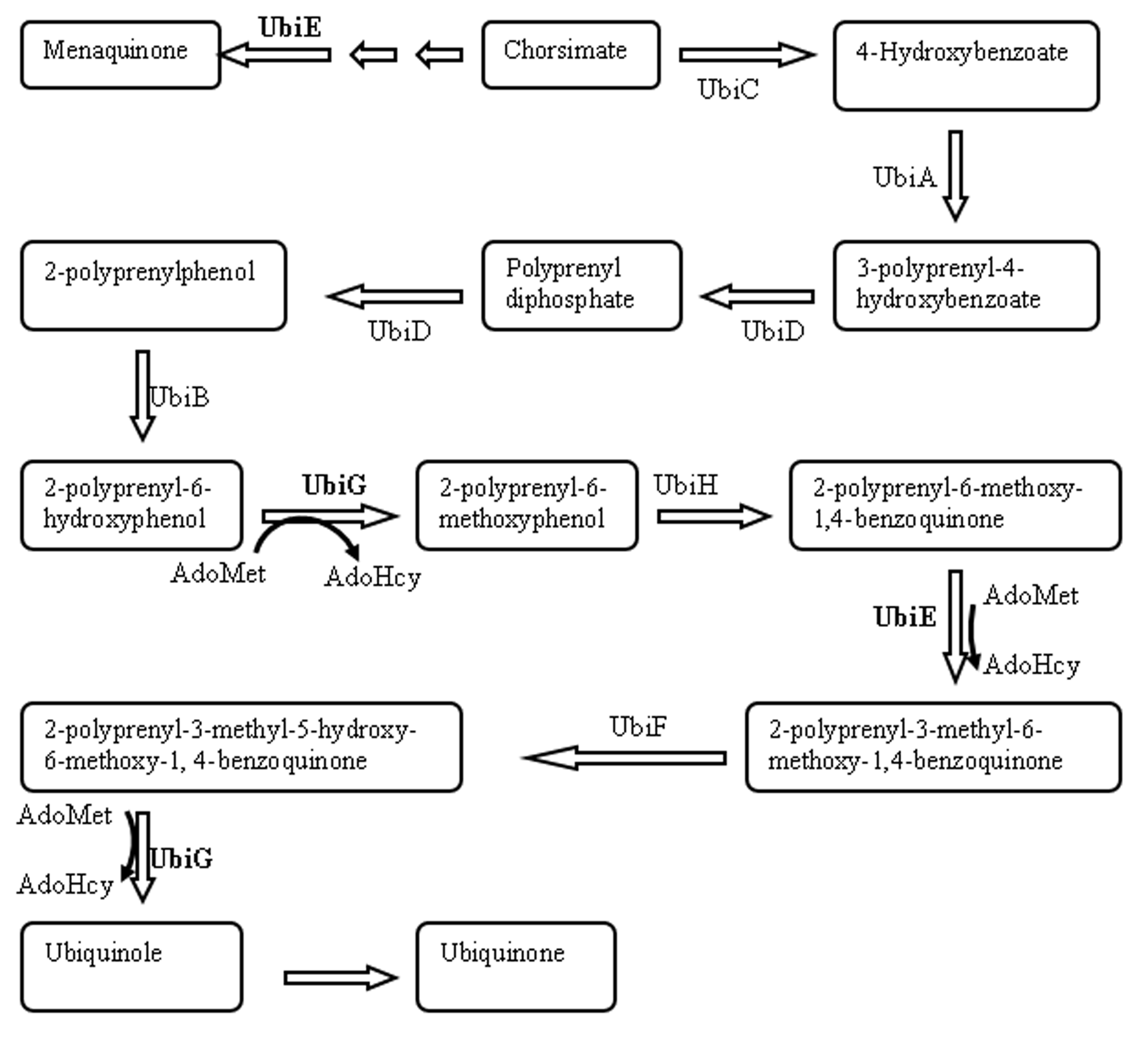

Supplement: Figure S2 — The schematic representation of the proposed biosynthesis of ubiquinone in bacteria. There are three methylation reactions in this pathway catalyzed by UbiE and UbiG. This figure is prepared based on the literature with possible intermediates and possible enzymes involved in this biosynthesis [27], [33]. Abbreviations used in this diagram are – UbiC: chorismate–pyruvate lyase; UbiA: 4-hydroxybenzoate polyprenyltransferase; UbiD: 3-polyprenyl-4-hydroxybenzoate carboxy-lyase; UbiB: ubiquinone biosynthesis monooxygenase UbiB; UbiG: ubiquinone biosynthesis AdoMet-dependent O-methyltransferase; UbiH: ubiquinone biosynthesis monooxgenase; UbiE: ubiquinone/menaquinone biosynthesis methyltransferase; and UbiF: ubiquinone biosynthesis monooxgenase. (TIF) [file pone.0027543.s002.tif]

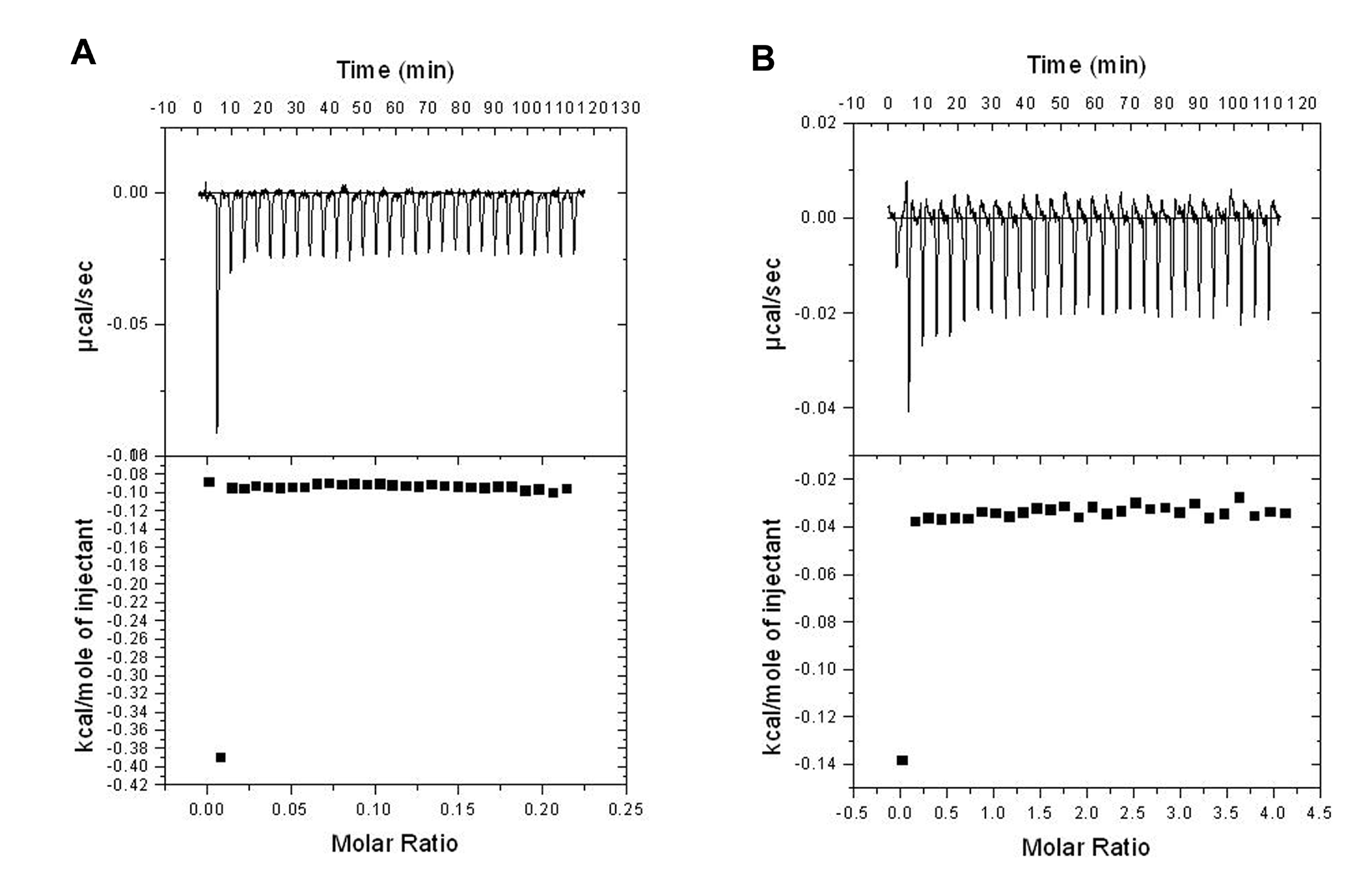

Supplement: Figure S3 — The ITC control experiments. A) Titration profile for AdoMet against buffer. A similar figure was obtained for AdoHcy titration against buffer. B) Titration of buffer against BT_2972 protein solution. (TIF) [file pone.0027543.s003.tif]

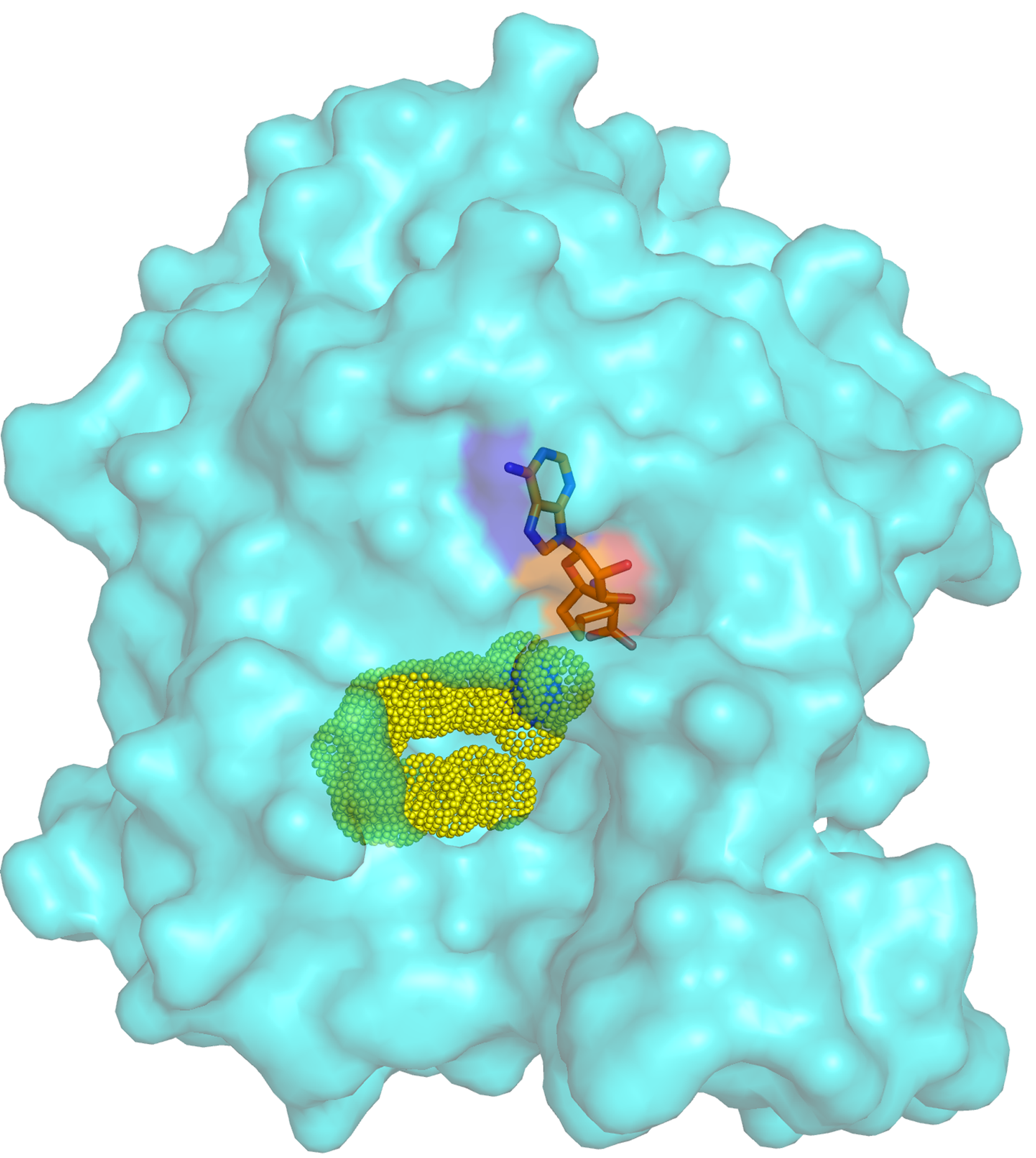

Supplement: Figure S4 — The molecular surface representation of the inferred substrate binding site with respect to the bound AdoHcy is shown as yellow dotted region on the surface of the BT_2972-AdoHcy complex. (TIF) [file pone.0027543.s004.tif]

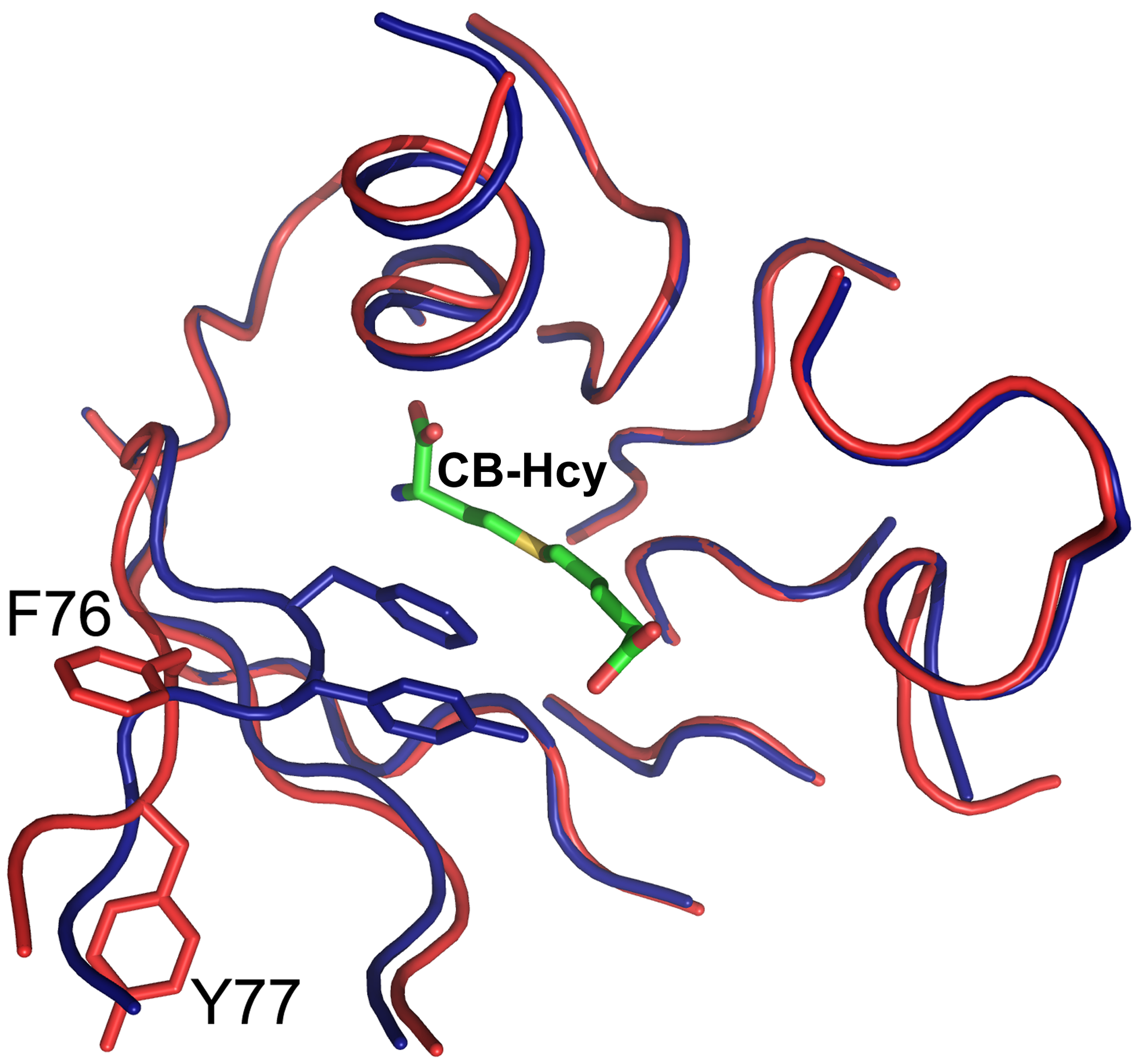

Supplement: Figure S5 — Figure shows the conformational change in betaine homocysteine S-methyltransferase upon substrate binding (PDB codes: 1UMY (from rat) and 1LT8 (from human)). The apo protein is shown in red and S-(D-carboxybutyl)-L-homocysteine (CB-Hcy) complex is in blue. The backbone and the side chain atoms of Phe76 and Tyr77 are shifted in the transition-state analog (CB-Hcy) complex in comparison with the apo structure. (TIF) [file pone.0027543.s005.tif]
